# Mr. Christie, on Vaccination

**Published:** 1805-02-01

**Authors:** Thomas Christie

**Affiliations:** Medical Superintendant General in Ceylon. Colombo


*Copy of a Letter from* Mr. Thomas Christie, *to* Dr. De Carro, &c. &c. &c. *of Vienna.*

Sir,

THE honourable Frederick North, Governor of Ceylon, has favoured me with the perusal of your work, on the progress of vaccination in Greece, Turkey and India, with the letter which accompanied it; and I have much pleasure in complying with His Excellency's commands, and your request, by giving some account of our success in that pursuit on this island.

From some passages of your work, I perceive you are acquainted with our progress in vaccination, up to the latter end of the year 1802; during the three l-st months of which, we inoculated nearly ten thousand people of all descriptions, in the different parts of this island.

Since that period, circumstances have been less favourable to our success, in extending the beneficial effects of this discovery, owing to the existence of a war in the interior of this island, which has called for the services in the field, of many medical men, who were most zealous promoters of vaccination, and has for a time thrown many of our own districts into a state of confusion and insurrection.

Notwithstanding these striking disadvantages, however, we have succeeded in keeping up a succession of patients with the true disease, at almost every considerable station on the island, since the first introduction of cow-pox on Ceylon in August 1802; and have, in all, extended our inoculations to upwards of twenty-one thousand persons.

On a tour round this island, for the purpose of inspecting the hospitals, and different medical establishments, which I have just finished, I was much graified to find from personal examination, that the disease existed in perfect purity at all the principal stations on Ceylon, and that the inhabitants, very generally, placed confidence in the preservative efficacy of the cow-pox, with the exception of the district of Jaffnapatnam, where the disease had become extinct, and vaccination got into disrepute, from the circumstance of a spurious disease having been introduced, after passing through which, several persons afterwards caught the small-pox.

This unfortunate circumstance is in a great degree, I believe, to be attributed to the practice having, at an early early period after its introduction, fallen into the hands of native practitioners, who, after a few proofs of its success, were satisfied if a puncture was made, and some irritation and inflammation produced. On inquiry, I found that they had frequently taken the matter at a very late period of the disease, and that in many instances an elevated pustule containing turbid matter was produced, and in others a high degree of inflammation succeeded by malignant ulceration; but all of them were equally supposed to have passed through the cow-pox; and if any of them afterwards were seized with small-pox, the practice of vaccination was thereby brought into disrepute.

I may here observe, that however simple the practice of inoculation may appear, I have seldom met with any of the natives possessed of that neatness, attention, perseverance, freedom from prejudice, and discrimination necessary for carrying on the practice with success, which is considerably more difficult with native subjects, in whom the colour of the skin renders the areola much less distinct than with Europeans, in whom it forms a diagnostic mark of the disease which can seldom be mistaken.

In the south-west side of the island, where we have been most successful in our inoculations, we have never trusted the disease out of the hands of the European practitioners, or young men, natives of the island, who have been bred at the European hospitals; and even with these last, I find it extremely difficult to convince them of the necessity of watching the disease through its whole progress, for they are apt to be deceived by the apparent simplicity of the operation, and too often inclined to think, that if a puncture is made, and some degree of inflammation follows, productive of a pustule, it is sufficient, without attending to the more nice distinctions of the disease.

Before I left Jaffna, in the begining of this month, I had two patients with the genuine disease brought from another station, Trincomale; and from these I inoculated the children of some of the principal families of the place, waited a fortnight till the disease had run its course in a regular manner, and then inoculated many of them with small-pox.—No disease was produced from the variolous inoculation; and at the time I left Jaffna, I had the pleasure to see, that confidence in the preservative efficacy of the cow-pox was restored to many of the most respectable inhabitants; and I then intrusted the care of the disease to Doctor Stuzer, Medical Superintendant, who, I doubt not, not, will be successful in gradually extending it through that district, which is the only one on Ceylon, which has not hitherto been essentially benefited by the effects of cow-pox inoculation.

By its benign influence, the small-pox of late has for the present been entirely banished from the popular districts of Colombo, Galle, and Matura, on the south-west side of the island; though for ten years previous to the introduction of the cow-pox, it had always, in a certain degree, prevailed in the Cittah of Colombo, and often occasioned the greatest ravages in the surrounding country.

With a view to lessen these ravages, Mr. North, with the attention which he always shews to the welfare and happiness of the people under his government, had, previous to the introduction of cow-pox, established hospitals for the reception of persons afflicted with small-pox, and confined inoculation to these hospitals, where it was promoted as much as possible; but notwithstanding every encouragement from government, and the greatest possible attention on the part of the medical gentlemen, only a few of the inhabitants were inclined to embrace the benefits of inoculation, and the natural small-pox continued to occasion considerable ravages, carrying off nearly one in three of all those who were seized with the disease. The appearance of small-pox in the more remote villages also often occasioned such a panic, that they were entirely deserted by the inhabitants; and the total stop to cultivation, and collection of the revenue, were the least distressing of the circumstances attending this desertion, as the wretched sick were too frequently forsaken by their nearest relations, and suffered to perish without assistance or nourishment.

It may be a fair calculation to suppose, that not one-half of the inhabitants of Ceylon escaped small-pox altogether; and as one-third of all those who were seized with it died, it would appear, that, that disease alone must have carried off, at least, one-sixth part of the whole population of this island.

The hospitals for the reception of patients with small-pox, and for the purposes of inoculation, were maintained at a very considerable expence for upwards of two years, and were certainly useful, by affording an asylum for persons afflicted with that disease, and also diminishing the risk of the infection being propagated in the natural form.

The benefit of inoculation, without injury to the community in general, was also extended to about four thousand sand persons; but even of these, one in forty or sixty, and sometimes still a larger proportion, died; whereas even the native practitioners, most inimical to cow-pox inoculation, have never pretended, that in any one instance on this island, it ever occasioned death, or even dangerous symptoms.

You will therefore with pleasure perceive, Sir, that there are few parts of the world, where the discovery of Dr. Jenner has, in so short a time, been more extensively beneficial to mankind than in Ceylon, as, notwithstanding the disadvantages attending a state of warfare, it has been communicated to upwards of twenty-one thousand people, in the short space of eighteen months, one-sixth part of whom, or three thousand and five hundred souls, would, agreeably to a fair and low calculation, have otherwise died of small-pox.

This reflection must be peculiarly gratifying to you, Sir, to whose exertions and attention the Indian world are obliged for the introduction of the cow-pox into these regions, probably some years before it would have otherwise reached us, by the circuitous route of the Cape of Good Hope; of which you will be convinced, when I inform you, that even so late as last year, an attempt was made, under the auspices of the British government and Dr. Jenner, to send out the vaccine to India with a detachment of Artillery, which sailed in the Windham and Walpole from England in February 1803. On their arrival on Ceylon in the June following, the matter sent from England was upon trial found to be effete, and either from a want of proper subjects, or from the matter having become inert, the surgeon of the Artillery, Mr. Morton, had not been able to produce the disease once in the course of the voyage, although he had made many attempts, agreeably to the instructions he had received, which were written by Dr. Jenner himself, and by Dr. Rollo, surgeon general of British Artillery.

Thus this attempt, under the most favourable auspices, to send the vaccine to India round by the Cape of Good Hope, would, as many others, have entirely failed; but on the arrival of the Artillery at Ceylon, we had the pleasure of communicating the cow-pox to such children as were born on the voyage, from your Cisalpine stock, which had reached us the preceding year, by Bagdad, Bassora, and Bombay.

You are informed that the disease was first produced in this island at Trincomalé, by threads received from

Bombay, in August, 1802; and from Trincomalé, it was speedily diffused throughout Ceylon, and also sent to Madras, and the other parts of the Coromandel coast; the threads sent there direct from Bombay having failed of success. From Madras, Dr. Anderson sent it to Bengal, in the ship Hunter, and it has thus been extended throughout the greater part of the Continent of India, and also sent from thence to the isles of France and Bourbon.

It appears however, that it had not reached Sumatra, or the other islands to the eastward, in the latter end of 1803, as by the arrival of a small vessel from Bencoolen last December, Mr. North received a dispatch from the Lieutenant Governor of Fort Marlborough, informing him, that every attempt to introduce it there, from dried matter, had hitherto failed of success, although the introduction of the cow-pox was most earnestly desired on Sumatra, as the ravages from small-pox were occasionally very great in that island.

His Excellency the Governor, with his usual zeal in the cause of humanity, immediately communicated his commands to me, to concert measures for the transmission of the vaccine, in an active state, to Sumatra.

Knowing the very uncertain effect of matter kept either in a dry or fluid state, for a considerable length of time, in this climate, I immediately, with the permission of His Excellency, procured six persons, who, there was every reason to believe, had not had either small-pox or vaccine, and embarked with them a medical assistant, who had long been in the habit of attending my practice, with directions to keep up the disease by successive inoculations, during the whole voyage, and to preserve a little matter from each patient. He was also charged with different packets of matter; and upon the whole, such precautions were taken, that I think there is almost no doubt of the objects being crowned with success, if the vessel reaches her destination.

On January 1, 1804, I inoculated the first of the six patients, with four punctures in different places, and on the same day the vessel was dispatched with her valuable cargo, and I believe was the first that ever undertook so long a voyage, which was calculated at four weeks, solely for the purpose of conveying the vaccine.

I have read with much attention and interest, the account given in your work of the grease and giardoni, as observed by M. La Font and Dr. Sacco, in Salonica and Italy,

Italy, and the hopes that Dr. Jenner and yourself entertain, that a similar disease will be found among the horses in India.

I most earnestly desire that your hopes may be realized; but assure you, that the subject has by no means been overlooked by me; since, as soon as I became acquainted with Dr. Jenner's opinion, with respect to the source of the cow-pox, and found that the dried matter sent from England to India failed of success, I immediately instituted inquiries with respect to the existence of the vaccine on the nipples of cows, or the true grease in the horses of this island.

In March, 1802, when I had the honour to accompany His Excellency the Governor in a tour round the island, this was one object of my inquiries; and by His Excellency's desire, I undertook solely for this purpose to visit the island of Delft, where there is an extensive stud of brood mares; but, after having been two days at sea, with a contrary wind, I was obliged to put back to Jaffnapatnam; I had however, afterwards, an opportunity of conversing with an English groom and a veterinary assistant, who had long attended the stud, and who assured me they had never seen any disease amongst the horses composing it similar to the true grease. The only one which approached to it was, what, from the name, I conceive may be the same mentioned by M. la Font, as the javart écrouelleux, the swelling of the fetlock joint, attended with fever, and followed by phagædenic ulceration, which often penetrated to the joint, and occasioned the death of the horse.

As soon as the cow-pox was established on this island, a medical officer was appointed at each of the principal stations of Colombo, Galle, Trincomalé, and Jaffnapatnam, with directions not only to promote the propagation of the vaccine, but also to watch over its preservation, and to supply the other practitioners with matter when required. It has seldom happened that the disease has become extinct at any of these stations; but when this, from any accident, has occurred, it has speedily been reproduced by matter, or vaccinated patients sent from one of the other stations. In this way we have been enabled to preserve the disease in perfect purity since August 1802, to the present period; and I think it is probable, that with care we may be enabled to keep up the infection for any length of time. I shall not, however, desist from my researches after the original source of the disease in the heels heels of horses, or nipples of cows, and shall recommend to the medical gentlemen on this island the inquiries suggested by you on this subject, as objects highly deserving of their most serious attention.

Allow me to conclude, Sir, by assuring you, that, with the other practitioners in India, I am highly sensible of the great obligations we owe to you, for your exertions in introducing the cow-pox into this country, which must prove the most important blessing ever bestowed on the eastern world, where the ravages committed by the small-pox were even still greater than in the more temperate climate of Europe.

I have the honour to be,
With the highest consideration,
Sir,
Your most obedient humble servant,
THOMAS CHRISTIE,
Medical Superintendant General in Ceylon.
(A true Copy.)

*Colombo, April* 23, 1804.

---

*Copy of a Letter from His Excellency the Hon.* FREDERIC NORTH, *to Dr. De Carro, of Vienna.*

MY DEAR SIR,

I am infinitely obliged to you for your very interesting work, and the kind letter which accompanied it. The enclosed, from my friend Mr. Christie, Superintendant General of Hospitals in this island, will give you a complete idea of the success of our efforts in vaccination. I am happy to add, that it has succeeded in Sumatra, as I have been informed by a letter which I received this morning from Bencoolen.

I have the honour to be,
With the greatest truth,
My dear Sir,
Your faithful humble servant,
FREDERIC NORTH.

*Colombo, June* 1, 1804.

